# Tick-borne Relapsing Fever Caused by *Borrelia hermsii,* Montana

**DOI:** 10.3201/eid0909.030280

**Published:** 2003-09

**Authors:** Tom G. Schwan, Paul F. Policastro, Zachary Miller, Robert L. Thompson, Todd Damrow, James E. Keirans

**Affiliations:** * Rocky Mountain Laboratories, National Institutes of Health, Hamilton, Montana, USA; † Group Health Cooperative of Puget Sound, Seattle, Washington, USA; ‡ Department of Public Health, State of Montana, Helena, Montana, USA; § Georgia Southern University, Statesboro, Georgia, USA

**Keywords:** tick-borne relapsing fever, *Borrelia hermsii*, *Ornithodoros hermsi*, Montana

## Abstract

Five persons contracted tick-borne relapsing fever after staying in a cabin in western Montana. *Borrelia hermsii* was isolated from the blood of two patients, and *Ornithodoros hermsi* ticks were collected from the cabin, the first demonstration of this bacterium and tick in Montana. Relapsing fever should be considered when patients who reside or have vacationed in western Montana exhibit a recurring febrile illness.

Tick-borne relapsing fever, caused by *Borrelia hermsii*, is endemic in the higher elevations and coniferous forests of the western United States and southern British Columbia, Canada (*1*). Although many multicase outbreaks of relapsing fever associated with *B. hermsii* and its tick vector, *Ornithodoros hermsi*, have been reported (*2*–*6*), none has been documented in Montana. Patients usually become ill after they have slept in cabins infested with spirochete-infected ticks that feed quickly during the night. The illness has an incubation period of 4 to >18 days and is characterized by recurring episodes of fever accompanied by a variety of other manifestations, including headache, myalgia, arthralgia, chills, vomiting, and abdominal pain (*1*). Relapsing fever is confirmed by the microscopic detection of spirochetes in the patient’s blood (Figure 1) (*7*).

**Figure 1:**
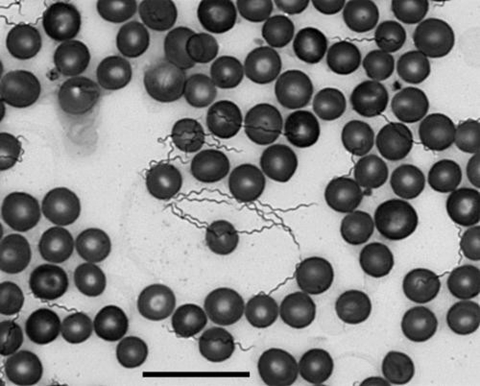
*Borrelia hermsii* in a thin smear of mouse blood stained with Wright-Giemsa stain and visualized with oil immersion bright-field microscopy (X600) for the confirmation of infection with relapsing fever spirochetes in humans and other animals. Scale bar = 20 μm.

In 1927, relapsing fever was diagnosed in a a 33-year-old man in Walla Walla, Washington, although his possible site of exposure was Montana (*8*). A specific location was not given, however, and spirochetes causing the illness were not identified. *Ornithodoros parkeri*, another tick vector of relapsing fever spirochetes in western United States, was collected during 1936 in Beaverhead County in southwestern Montana, and an undisclosed number of these ticks transmitted *Borrelia parkeri* to one mouse in the laboratory (*9*). If relapsing fever had occurred in Montana, *B. parkeri* transmitted by *O. parkeri* would have been the likely etiologic agent (*9*,*10*).

In summer 2002, a multicase outbreak of relapsing fever associated with a privately owned cabin occurred in western Montana. Spirochetes were isolated from two patients and identified as *B. hermsii*, and this spirochete’s tick vector, *O. hermsi*, was collected from the cabin where the patients slept. This is the first multicase outbreak of tick-borne relapsing fever in Montana and the first report of *B. hermsii* and *O. hermsi* in the state, thereby documenting the risk of this infection beyond the geographic range known previously within the United States.

## The Study

From July 30 to August 20, 2002, a total of 5 persons in a group of 20 became ill with symptoms consistent with tick-borne relapsing fever during or following their visit to western Montana (Table). The common site of exposure was a cabin on the south shore of Wild Horse Island (47°50′30” N; 114°12′30” W) in southwest Flathead Lake, Lake County, Montana. The 875-hectare island became a state park in 1978, although 56 privately-owned properties exist, many of which have cabins. No one lives permanently on the island, and camping overnight (by day visitors to the island) is not allowed. The island is approximately 4.6 km wide from east to west and 3.2 km wide from north to south; its elevation varies from 881 m at the shoreline to its highest point of 1,141 m. The island is separated from the mainland by 2.0 km to the south and 2.4 km to the north. The habitats include Ponderosa Pine and Douglas Fir forests, native grassland, and steep rocky outcroppings. Red squirrels (*Tamiasciurus hudsonicus*) and deer mice (*Peromyscus maniculatus*) are abundant.

**Table:** Summary of relapsing fever patients exposed in western Montana during July–August, 2002

Case no.	Patient age (y)^a^	Sex	Onset	Signs/Symptoms	Initial blood smear	Final laboratory results
1	54-	M	July 30^b^	38.9°C–39.4°C temperature, rash, headache, myalgia, arathralgia	Spirochete negative	Seropositive by IFA^c^ and Western blot
2	5	F	August 10	40.6°C temperature., vomiting, diarrhea, headache, myalgia	Spirochete positive	Mouse inoculation positive; isolation of *B. hermsii*
3	43	M	August 16	Fever, headache, myalgia, arthralgia	Spirochete positive	Mouse inoculation positive; isolation of *B. hermsii*
4	43	F	August 30	38.9°C–39.4°C temperature, vomiting, diarrhea, headache	Spirochete negative	Mouse inoculation negative; no isolation
5	13	M	August 11	40.6°C temperataure, vomiting, headache	Spirochete positive	Mouse inoculation and isolation not attempted

^a^Confirmed cases with demonstration of spirochetes in blood; presumptive cases with appropriate manifestations but no spirochetes detected. ^b^This patient relapsed on August 6. ^c^IFA, immunofluoresence assay

On July 22, the first of four related families arrived at the cabin, and on July 25, a 54-year-old man (case 1, Table) entered the east end of the attic and removed nest material that had accumulated there. He slept at night and napped during the day in one of two bedrooms located immediately under the area of the attic where the nest material had been partially removed. On July 30, he became ill with fever, headache, arthralgia, myalgia, and rash, and 2 days later he visited the emergency room of a local hospital but a diagnosis was not made. Over the next several days he improved, and on August 6, he and his family began driving back to their home in Seattle, Washington. During the trip, he relapsed with another febrile episode. That evening, he was taken to the emergency room of a Seattle hospital and admitted early the next morning. On the basis of his history, a diagnosis of relapsing fever was considered, although spirochetes were not detected in the blood.

Three additional families (17 persons) arrived at the cabin on July 31 and on August 5 and departed on August 8 and 9. One family of five returned to their home in Seattle, and three of them became ill on August 12, 17, and 20 (cases 2–4). Relapsing fever was suspected immediately, and spirochetes were detected in Wright-stained blood smears from two patients (cases 2, 3). On August 10, a family of six returned to St. Louis, Missouri, where a 13-year-old boy (case 5) became ill the next day. On August 12, he was taken to an emergency room and to his pediatrician the following day. His mother communicated with the family in Seattle, where a young girl (case 2) was ill, and spirochetes had been detected in her blood. This discovery led to the detection of spirochetes in a blood smear from the boy. All patients had fever and other clinical manifestations consistent with tick-borne relapsing fever (Table). They were all treated with doxycycline, and all recovered with no subsequent relapses.

**Figure 2:**
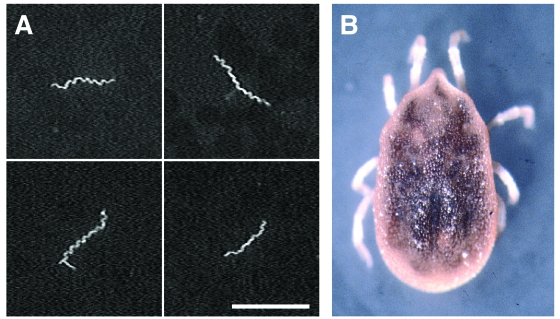
(A) *Borrelia hermsii* in the blood of one patient (case 3) stained with rabbit hyper-immune serum and anti-rabbit fluorescein isothiocyanate. Scale bar = 20 μm. (B) An *Ornithodoros hermsi* nymph collected from the attic of the cabin. The length of the tick is 3.0 mm, excluding the legs.

Blood smears from three of the Seattle patients (cases 2–4) were prepared and stained separately with monoclonal antibodies H9724, which recognizes all known species of *Borrelia* (*11*), and H9826, which is specific for *B. hermsii* (*12*), and rabbit hyperimmune serum to *B. hermsii* (Figure 2A). Indirect immunofluorescence assays (IFA) and microscopic analysis demonstrated spirochetes from two patients (cases 2, 3) that were reactive with all antibodies, which identified these bacteria as *B. hermsii*. Blood from the third patient (case 4) was negative for spirochetes with all antibodies. EDTA-treated whole-blood samples from these patients were injected intraperitoneally into mice, and the two samples positive by microscopic examination also produced detectable levels of spirochetemia in mice. Whole blood obtained from the infected mice was injected into modified Kelly’s medium (BSK-H supplemented with 12% rabbit serum; Sigma-Aldrich Corp., St. Louis, MO), and spirochetes that originated from two patients were isolated.

A convalescent-phase serum sample from the first case-patient (case 1) was collected 55 days after the onset of his illness. This sample was examined by IFA with whole cells of *B. hermsii* (*13*) and by immunoblot with a whole-cell lysate of *B. hermsii* and recombinant GlpQ (*13*). The patient’s IFA titer to *B. hermsii* was positive at 1:1,024, and the sample was positive by immunoblot at 1:100 dilution.

The five persons with confirmed or presumptive relapsing fever slept in two adjacent bedrooms in the east end of the cabin under the attic where animal nest material had been partially removed. People who slept only on the outside porch or in other bedrooms did not become ill. On August 24, 2002, the two east bedrooms were examined for ticks, but none were found. The remaining nest material was collected from the attic and taken to Rocky Mountain Laboratories. During the next several weeks, the material was processed with two small Berlese extraction funnels, which separate live arthropods from nonliving debris. Fourteen *O. hermsi* were recovered, including 1 larva, 10 nymphs, 2 males, and 1 female (Figure 2B). The postlarval stages of *O. hermsi* are very similar to those of *O. sparnus,* which parasitizes woodrats and deer mice in Utah and Arizona, but the latter species is an incompetent vector of *B. hermsii* (*14*,*15*). The larva collected from the cabin displayed morphologic characteristics consistent with *O. hermsi*. Voucher specimens (one nymph, one larva) of *O. hermsi* collected at the study site were deposited in the U.S. National Tick Collection, Georgia Southern University, under accession number RML 123385. The 12 remaining ticks were allowed to feed on a laboratory mouse to determine whether they were infectious. The blood of the mouse did not become spirochetemic during the 10 days after tick bite. These ticks were not examined for infection by other methods and were kept alive to establish a laboratory colony.

On June 21, 2003, the attic, utility room, and bedrooms where the infected persons slept were treated with an over-the-counter insecticide-acaricide (Ortho Indoor Insect Fogger, The Ortho Group, Columbus, OH). Sentinel *O. hermsi* ticks (late stage nymphs and adults) from a laboratory colony were confined in open flasks in one treated bedroom (46 m^3^) and a family room that was not treated to examine the efficacy of treatment. After the 4-hour application with two 141-gm cans of fogger, all 54 ticks in the treated bedroom were dead, whereas all 52 ticks in the untreated room were alive.

## Discussion

Tick-borne relapsing fever caused by *B. hermsii* is acquired only within the geographic range of its specific tick vector, *O. hermsi*. This tick has been found in southern British Columbia, Washington, Idaho, Oregon, California, Nevada, Colorado, and the northern regions of Arizona and New Mexico (*2*,*4*,*16*). As this and other outbreaks demonstrate, patients often become ill after they leave disease-endemic areas where they were bitten by infectious ticks (*2*,*6*). One patient (case 1) remained untreated early in his illness in spite of seeking medical attention at a hospital near the site of exposure.

The cabin where the patients were infected has been owned by the same family for nearly 40 years. None of the members of the four related families questioned recalled any prior illnesses consistent with what they experienced with this outbreak of relapsing fever. The event that appears to have instigated this outbreak was the partial removal and disturbance of animal nest material in the east end of the attic. Some ticks presumably fell through the spaces between the ceiling boards to the two bedrooms below. The boy (case 5) slept all but part of one night on the porch, but during the night of August 6 a thunderstorm forced him indoors, and he moved to the front east bedroom. His onset of illness in St. Louis was on the afternoon of August 11, which equates to an incubation period of approximately 4.5 days. The incubation periods for the others were estimated at 5 to 15 days.

The animals that maintained the enzootic cycle with *B. hermsii* and *O. hermsi* in the cabin are unknown. Red squirrels are highly susceptible to infection with *B. hermsii* (*17*), are important hosts for these ticks (*1*), and were abundant in the forest surrounding the cabin. However, no evidence of squirrels was found in the cabin. Deer mice were routinely in the cabin, and the owners used poison bait stations to control the indoor population. One dead mouse was found near the cabin, and two carcasses were in the attic material that had been removed on July 25. American robins (*Turdus migratorius*) had nested in the attic, and two dead robin chicks were found in the material collected from the attic on August 24. Recently, a *B. hermsii*–like spirochete was implicated in the death of a northern spotted owl (*Strix occidentalis*) in Kittitas County, Washington (*18*), and many years ago, 26 *O. hermsi* were collected from the nest of a bluebird (either *Sialia mexicana* or *S. currucoides*) in Summerland, British Columbia (*19*). The role of birds in perpetuating relapsing fever spirochetes and their tick vectors in nature is worthy of further investigation. A serologic survey of red squirrels and deer mice in the vicinity of the cabin for immunologic evidence of exposure to *B. hermsii* might also help explain the enzootic involvement of these rodents.

This outbreak demonstrated for the first time that *B. hermsii* and its tick vector *O. hermsi* exist in Montana and caused multiple cases of relapsing fever. Owners of cabins in the vicinity of where the outbreak occurred met with the Montana state epidemiologist and received information regarding the epidemiology and prevention of tick-borne relapsing fever. Although the outbreak was localized, a large area of western Montana has the appropriate ecologic parameters to support enzootic cycles that provide the potential for relapsing fever caused by *B. hermsii* to occur. A diagnosis of relapsing fever should therefore be considered when patients who have resided or vacationed in western Montana seek treatment for a recurrent febrile illness.
